# Thigh Compartment Syndrome Following Physician-Modified Fenestrated Endograft Aneurysm Repair

**DOI:** 10.31486/toj.25.0026

**Published:** 2026

**Authors:** Alexander Crowley, W. Reed Bigham, Mila Scheinberg Hurley, Godfrey Ross Parkerson, Ross Dunbar

**Affiliations:** ^1^ Department of Orthopedic Surgery, Ochsner Clinic Foundation, New Orleans, LA; ^2^ Department of Vascular Surgery, Ochsner Clinic Foundation, New Orleans, LA; ^3^ The University of Queensland Medical School, Ochsner Clinical School, New Orleans, LA

**Keywords:** *Compartment syndromes*, *critical limb-threatening ischemia*, *fasciotomy*, *reperfusion injury*, *thigh*

## Abstract

**Background:**

Thigh compartment syndrome (TCS), a rare but serious complication of vascular procedures, often leads to severe morbidity. Unlike leg compartment syndrome, TCS can present insidiously, delaying diagnosis and treatment. While most cases of TCS are trauma-related, vascular causes such as ischemia-reperfusion injury have been reported.

**Case Report:**

A 67-year-old male with coronary artery disease, chronic obstructive pulmonary disease, and a 50 pack-year smoking history underwent physician-modified fenestrated endograft repair of an unruptured thoracoabdominal aortic aneurysm. The procedure, performed via bilateral common femoral artery access, required upsizing the right femoral sheath to 26 French from the original 22 French because of delivery challenges. Postoperatively, the patient developed severe right lower extremity pain and loss of sensation, prompting emergent 4-compartment fasciotomy for leg compartment syndrome. Hours later, he developed new-onset thigh pain, paresthesia, and restricted movement, leading to the diagnosis of TCS. Emergent 3-compartment thigh fasciotomy was performed. The patient's postoperative course was complicated by anterior cord syndrome, paraplegia, and end-stage renal disease resulting from severe rhabdomyolysis. One year later, the patient remained on dialysis with chronic fasciotomy wounds.

**Conclusion:**

This case highlights the rare occurrence of TCS following physician-modified fenestrated endograft and the potential for serious complications. Given the increased risk associated with prolonged procedures and large-diameter sheaths (≥20 French), close postoperative monitoring is crucial. Early recognition and timely intervention are key to improving patient outcomes.

## INTRODUCTION

Thigh compartment syndrome (TCS) is a rare orthopedic emergency that is associated with risks of long-term functional deficits, morbidity, and mortality. TCS can have a subtle and gradual presentation, unlike the acute onset of leg compartment syndrome, that may contribute to delayed recognition and management.^1^ A retrospective study found that TCS was present in only 6.6% of compartment syndrome cases in their cohort and occurs in just 0.3% of trauma patients.^2^ TCS is a specific form of acute compartment syndrome that affects the myofascial compartments of the thigh. Characterized by elevated pressure within these compartments, acute compartment syndrome leads to decreased perfusion, potentially resulting in irreversible myonecrosis and neurovascular injury. Because of the rarity and often insidious onset of TCS, diagnosis can easily be delayed or altogether missed. While most cases of TCS are trauma-related, atraumatic and vascular etiologies have also been described.^2^

We present a case of TCS secondary to ischemic-reperfusion injury with delayed presentation following physician-modified fenestrated endograft (PMEG).

## CASE REPORT

A 67-year-old male with a medical history of coronary artery disease, chronic obstructive pulmonary disease (COPD), and a 50 pack-year smoking history presented to the vascular surgery clinic with a known unruptured abdominal aortic aneurysm. Outpatient computed tomography revealed aneurysmal degeneration of the proximal supraceliac and paravisceral abdominal aorta, with a maximum diameter of 5.7 cm at the T12 level of the aortic hiatus (Figure). Because of his COPD, the patient was not healthy enough to undergo open thoracoabdominal aneurysm repair, and because no endovascular graft was commercially available for treatment of thoracoabdominal aneurysm, a PMEG repair was performed.

**Figure.**
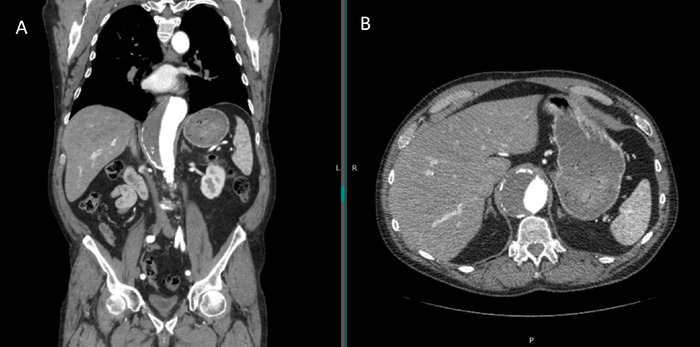
(A) Sagittal and (B) axial computed tomography images show aneurysmal degeneration of the proximal supraceliac and paravisceral abdominal aorta, with a maximum diameter of 5.7 cm at the level of the aortic hiatus (T12 vertebra).

The procedure involved bilateral common femoral artery access with precannulation wires placed via left axillary artery cutdown. Once the endograft was modified on the back table, it was repackaged into the sheath but could not be advanced through the original sheath for delivery. The original 22 French (Fr) sheath was removed, and a larger 26 Fr sheath was used to deliver the device. Because of the complexity of the case and the complications of graft delivery, the procedure lasted 8 hours. After the endograft was placed, the patient was transferred to the surgical intensive care unit in stable condition.

Two hours postoperatively, the patient began reporting severe right lower extremity pain and loss of sensation distal to the knee. On examination, he had palpable dorsalis pedis and posterior tibial pulses. However, severe pain with passive dorsiflexion and plantarflexion of the right ankle was noted in addition to incompressible anterior, lateral, and superficial posterior compartments of the right leg. The patient was taken emergently to the operating room where Vascular Surgery performed a 4-compartment release via a 2-incision approach.

Five hours postoperatively, Orthopedics was consulted to evaluate the patient's right thigh because of new-onset paresthesia and decreased active range of motion. Tense anterior and posterior thigh compartments were noted on examination, and the patient demonstrated severe pain with passive knee range of motion. TCS was diagnosed, and the patient was taken for emergent 3-compartment release of the thigh.

The patient's postoperative course was complicated by bleeding from his thigh fasciotomy wound, rhabdomyolysis, acute renal failure, and anterior cord syndrome. In addition to the extensive thoracic aortic endograft coverage, the patient's risk of anterior cord syndrome was increased by the bleeding from the thigh fasciotomy wound, which led to anemia and relative hypotension. The patient developed acute renal failure as a result of the rhabdomyolysis. The patient's renal function did not recover, and 1 year postoperatively, he remained on hemodialysis with end-stage renal disease.

## DISCUSSION

Compartment syndrome is a medical condition characterized by increased pressure within a closed fascial compartment containing muscles, nerves, blood vessels, and other vital structures. The elevated pressure within the fascial compartment compresses blood vessels and impedes blood flow, leading to decreased oxygen and nutrient delivery to tissues and resulting in tissue ischemia and eventual necrosis. Etiologies that cause increased pressure, such as trauma, fractures, crush injuries, and soft tissue injuries, account for >75% of compartment syndrome cases.^3^ Other notable causes include burns, vascular and reperfusion injuries, constrictive bandages or casts, and prolonged limb compression. Vascular etiologies are widely regarded to be a less frequent cause of compartment syndrome.^3^ The anterior compartment of the leg is the most common site of compartment syndrome. Less frequently, compartment syndrome can occur in the forearm, abdomen, thigh, and gluteal region.^4^

Common signs and symptoms for compartment syndrome presentation can be remembered by the 6 Ps: (1) pain, (2) poikilothermia, (3) paresthesia, (4) paralysis, (5) pulselessness, and (6) pallor.^5^ The pain is often described as out of proportion to the injury, is typically not relieved by rest or pain medication, and may increase with passive movement or stretching of the muscle.^3^ Observing for skin changes, such as swelling and color change, palpating over the affected compartment for tension and tenderness, checking pulses, and evaluating for motor and sensory function are vital aspects of assessing for possible compartment syndrome.

Measurement of compartment pressure, although not required, can aid in the diagnosis. Intracompartment pressure measurements can assist in the diagnosis of acute compartment syndrome. A delta pressure (diastolic blood pressure minus intracompartmental pressure) of ≤30 mm Hg is commonly used as a threshold for considering fasciotomy, whereas a delta pressure >30 mm Hg is generally considered safe to rule out the diagnosis of acute compartment syndrome.^6^

TCS requires prompt diagnosis and surgical treatment to prevent severe outcomes, including permanent neuromuscular dysfunction, limb loss, and systemic complications such as rhabdomyolysis and renal failure.^2^

Lower extremity compartment syndrome following PMEG is rare; the only case we found in the literature involved the leg compartment rather than the thigh.^7^ The pathophysiology in our patient was likely multifactorial, including ischemia-reperfusion injury, prolonged operative time, and the use of a large-diameter (26 Fr) sheath. Operators should be mindful of the placement of the sheath, particularly when larger sheath sizes are used. Parking the top of the sheath tip within the external iliac artery can reduce thigh ischemia by allowing flow through internal iliac to profunda branch collateral pathways. A sheath parked in the common iliac artery and obstructing flow through this collateral pathway can lead to more severe thigh ischemia and subsequent reperfusion injury. Ischemia-reperfusion injury can cause endothelial dysfunction, capillary leakage, and tissue edema, exacerbating intracompartmental pressure and leading to muscle necrosis.^8^ A large-diameter femoral sheath may also contribute to local vascular injury, further compromising limb perfusion.

A major challenge in diagnosing TCS is the often insidious presentation, which can delay intervention.^1^ Unlike leg compartment syndrome that frequently presents with the classic 6 Ps as previously mentioned, TCS may initially manifest as vague thigh discomfort or weakness, making clinical suspicion crucial.^1^ In our patient, despite timely leg fasciotomy, the delayed onset of thigh symptoms underscored the need for continuous postoperative reassessment.

This case also underscores the systemic consequences of compartment syndrome, as our patient developed anterior cord syndrome and end-stage renal disease as a result of severe rhabdomyolysis. The extent of these complications highlights the importance of early recognition and intervention.

Given the growing use of advanced endovascular techniques and large-caliber sheaths, vascular and orthopedic surgeons must maintain a high index of suspicion for compartment syndrome during the postoperative period. Prolonged PMEG procedures warrant close monitoring for signs of limb ischemia, and the threshold for fasciotomy should be low in patients with evolving symptoms. Additionally, routine postoperative assessment should extend beyond the lower leg to include the thigh, particularly in cases involving prolonged ischemia or large femoral access sites.

Our patient's case was complicated by rhabdomyolysis, acute renal failure, and subsequent anterior spinal artery syndrome. This case highlights the rarity of TCS in the setting of vascular intervention.^1,9^ Given the limited data available on TCS as a complication of vascular surgery, a multi-institutional retrospective review would be valuable for identifying risk factors, improving early recognition, and guiding postoperative monitoring protocols in high-risk patients.

## CONCLUSION

This case underscores the complexity of diagnosing TCS caused by vascular injury and its insidious onset in comparison to leg compartment syndrome. Lower extremity compartment syndrome following PMEG is uncommon, with only one case reported in the literature, but can result in severe complications as demonstrated in our patient. Patients undergoing prolonged PMEG procedures should be monitored closely in the postoperative period, especially if large-diameter sheaths (≥20 Fr) are used intraoperatively. Increased awareness and early intervention are critical in improving outcomes for this severe complication.

## Notes

*This article meets the Accreditation Council for Graduate Medical Education and the American Board of Medical Specialties Maintenance of Certification competencies for Patient Care and Medical Knowledge.*
